# Multistate Outbreak of Human *Salmonella* Poona Infections Associated with Pet Turtle Exposure — United States, 2014

**DOI:** 10.15585/mmwr.mm6429a7

**Published:** 2015-07-31

**Authors:** Colin Basler, Lyndsay Bottichio, Jeffrey Higa, Belinda Prado, Michael Wong, Stacey Bosch

**Affiliations:** 1 Epidemic Intelligence Service, CDC; 2 Division of Foodborne, Waterborne, and Environmental Diseases, National Center for Emerging and Zoonotic Infectious Diseases, CDC; 3 California Department of Public Health; 4 City of Long Beach Department of Health and Human Services, Long Beach, California

In May 2014, a cluster of human *Salmonella* Poona infections was identified through PulseNet, the national molecular subtyping network for foodborne disease surveillance. Historically, this rare serotype has been identified in multiple *Salmonella* outbreaks associated with pet turtle exposure and has posed a particular risk to small children (1,2). Although the sale and distribution of small turtles (those with carapace [upper shell] lengths <4 inches [<10.2 cm]) is prohibited by federal law, they are still available for legal purchase online for “bona-fide” scientific, educational, or exhibition purposes, other than use as pets (3). In addition, small turtles are still available for illegal purchase through transient street vendors, at flea markets, and at fairs.

During April 26–September 22, 2014, a total of 40 persons infected with *Salmonella* Poona pulse-field gel electrophoresis (PFGE) pattern JL6X01.0055 (the outbreak strain) were reported from 12 states. Patients ranged in age from <1 to 75 years (median = 5 years); 16 (40%) patients were aged ≤1 year, and 14 (35%) were female. Among 29 ill persons for whom information about hospitalization was available, eight (28%) were hospitalized; no deaths were reported. Among 28 ill persons who were interviewed, 13 (46%) reported exposure to turtles. Three ill persons reported the size of the turtles, and all identified turtles <4 inches in length. The outbreak strain was isolated from a pet turtle in a California patient’s home. Turtles had been obtained from several types of locations, including a carnival and a fair. The transient nature of turtle vendors hampered the traceback investigation. No other common food or animals were identified during the course of the investigation.

This outbreak demonstrates that turtles remain a source for human *Salmonella* infections, especially for young children. Because 40% of ill persons were infants aged ≤1 year and were unlikely to directly handle pet turtles, the potential role of indirect transmission in turtle-associated salmonellosis outbreaks should be considered. Turtles in the home could lead to environmental contamination with *Salmonella* bacteria and result in human illness. Educational campaigns directed toward parents of young children, in conjunction with the federal turtle ban, might help to prevent future turtle-associated salmonellosis outbreaks.
